# Accuracy of Dental Replica Models Using Photopolymer Materials in Additive Manufacturing: In Vitro Three‐Dimensional Evaluation

**DOI:** 10.1111/jopr.12928

**Published:** 2018-07-02

**Authors:** Su‐Jin Jin, Dong‐Yeon Kim, Ji‐Hwan Kim, Woong‐Chul Kim

**Affiliations:** ^1^ Department of Public Health Science, Graduate School Korea University Seoul Korea; ^2^ Department of Dental Laboratory Science and Engineering, College of Health Science Korea University Seoul Korea

**Keywords:** Additive manufacturing, dental replica model, photopolymer, precision, trueness

## Abstract

**Purpose:**

To evaluate the accuracy (trueness and precision) of dental replica models produced by using photopolymer materials in additive manufacturing.

**Materials and Methods:**

A complete arch model was scanned using an extraoral scanner (Identica Blue) and established as reference. For the control group, 10 stone models were acquired through the conventional method from the reference model. For the experimental groups, digital data were acquired using an intraoral scanner (CEREC Omnicam), and 10 stereolithographic apparatus (SLA) models and 10 PolyJet models were made. All models were scanned with an extraoral scanner. Three‐dimensional analysis software was used to measure differences between the 3D scanned images in root mean square values. The ISO‐5725‐1 specification was followed to measure trueness and precision between two 3D scanned data. Trueness was calculated by overlapping scanned data with the reference model and precision by performing pairwise intragroup comparisons. Also the ratio of region out of tolerance (> ±50 μm) was measured. One‐way ANOVA and Tukey's post hoc analysis were applied.

**Results:**

There was no statistically significant difference in trueness between the stone and the SLA models (*p > *0.05). Dental replica models using photopolymer materials showed statistically significantly better precision than that of the stone model (*p < *0.05). Regarding tolerance, no statistically significant difference was observed between the stone and the SLA models (*p > *0.05).

**Conclusions:**

Although the dental replica models using photopolymer materials did not show better trueness than the conventional stone models, there was no significant difference between the SLA and the stone models. Concerning precision, dental replica models using photopolymer materials presented better results than that of the conventional stone models. In sum, dental replica models using photopolymer materials showed sufficient accuracy for clinical use.

Computer‐aided design/computer‐aided manufacturing (CAD/CAM) has introduced revolutionary changes not only in many industries but also in dentistry, allowing for a better clinical experience and quality.^1^, ^2^, ^3^ In dentistry, manually created stone models were previously used, and they required a longer manufacturing time and greater caution to avoid breaks or deformations; however, the use of CAD/CAM in dentistry eases all steps in the dental treatment process, from diagnosis to treatment. Additionally, it does not require the storage of physical models, as it is possible to produce them from the scanned data whenever needed.^4^

Among these computer‐aided approaches, additive manufacturing (AM) methods are being actively developed, given their capability to easily manufacture complicated models.^1^ Of these AM methods used for dental replica models, the stereolithographic apparatus (SLA) method and PolyJet (photopolymer jetting) method using photopolymer materials, are known to have good accuracy.^1^, ^5^ The SLA method builds 3D shapes by accumulating cured layers of liquid resin in a water bath by laser beam,^3^ while the PolyJet method mimics the mechanism of an inkjet printer, using liquid photopolymers instead of ink and solidifying them with UV light, by the photocuring process.^1^

Although these two AM methods for dental replica models using photopolymer materials have various advantages over the conventional method, AM material shrinkage during the photocuring process can introduce differences from the reference model,^6^ and the stair‐step effect, which represents uneven surfaces due to the accumulation of layers, causes uneven surfaces and produces less accurate models.^1^, ^7^, ^8^ To apply AM methods in clinical use, the accuracy of existing AM technology with these limitations must be validated.

In this study, the ISO standard^9^ for the accuracy of dental models was applied. This standard specifies that accuracy comprises two factors: trueness as quantified by systematic errors and precision as quantified by random errors. Trueness is the measure of the deviation from the given reference, and precision is the measure of the deviation from repeated measurements in the same group.

The aim of this study was to measure the trueness and the precision of AM models using photopolymer materials and to compare them with conventional stone models. The null hypothesis of this study was that trueness and precision of these AM models using photopolymer materials were not significantly different from those of the conventional stone model.

## Materials and methods

### Reference model and conventional models

**Figure 1:**
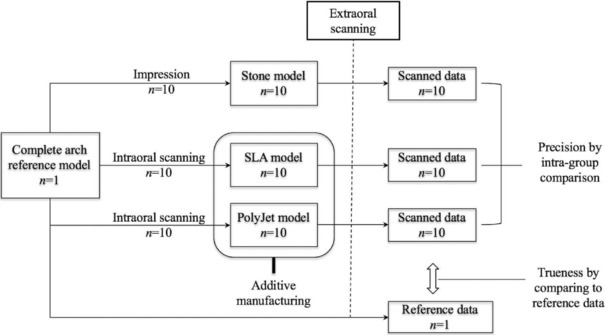
Schematic diagram of the experiment performed in this study.

A complete‐arch model (ANKA‐4 V CER; Frasaco, Tettnang, Germany) was used as the reference model, which includes removable upper and lower model jaws with the Frasaco click system. To obtain the conventional stone models, heavy‐ and light‐body silicone impression materials (Aquasil Ultra XLV and Rigid; Dentsply Caulk, Milford, DE) were used in a metal stock tray, and 10 impressions of the reference model were obtained. The manufacturer's recommendation for the duration for setting the impressions was applied. To increase the quality of the cast models, a wetting agent was sprayed three times inside the impression, followed by pouring type IV stone (Jade stone; Whip Mix Corp, Louisville, KY) into the impression body and hardening for 45 minutes. The hardened stone models were kept at room temperature for 48 hours (Fig 1).

### AM models

For the input data of AM modeling, an intraoral scanner (CEREC Omnicam; Sirona, Bensheim, Germany) was used on the reference model to obtain surface tessellation language (STL) files (*n = *10) using the manufacturer's certified software (CEREC Connect Software 4.3; Sirona) with recommended scanning path. With these 3D data, SLA (ProJet 6000; 3D Systems, Rock Hill, SC) and PolyJet (ProJet 3500 HD Max; 3D Systems) were used to manufacture 10 AM models each. For these two AM systems, the manufacturer's recommended settings were applied. The material used for SLA was photocurable liquid resin (VisiJet SL Clear; 3D Systems), and the material used for PolyJet was acrylic polymer (VisiJet M3‐X‐Rigid White; 3D Systems).

### Reference data and scanned data acquisition from the models

The reference model, 10 conventional stone models, and 10 SLA and 10 PolyJet models were scanned using a bench top extraoral scanner (Identica Blue; Medit, Seoul, South Korea) to obtain the corresponding scanned data as an STL file, one reference data, and three groups (stone, SLA, PolyJet models) of 10 scanned data. These manufactured models were measured within the first 7 days of manufacturing. Before scanning the 20 AM models, an imaging powder (VITA CEREC powder scan spray; VITA, Bad Säckingen, Germany) was applied onto the surface of these models to obtain a more uniform reflection.^10^

### 3D analysis

The reference data and all scanned data were prepared for the 3D analysis by removing the gingival area approximately 1 mm away from the cervical line. The reference data and the scanned data were superimposed with a best‐fitting method using a 3D analysis software (Geomagic Verify 2015; 3D Systems) to measure trueness and precision values. Trueness was determined by comparing the reference data with three groups of the scanned data (stone, SLA, and PolyJet model groups [*n = *10 in each group]), and precision was calculated by comparing all pairs in each scanned data group (*n = *45 in each group). The root mean square (RMS) value was used to quantify the trueness, and precision and was calculated as follows:

RMS =∑(x^i−xi)2∑(x^i−xi)2n

where ${\hat{x}}_{i}$ is the measured reference data at point *i*, $x_{i}$ is the measured scanned data at point *i*, and *n* is the total number of points.

Using 3D deviation image analysis, color‐coded image data were obtained to visualize the differences between the reference and the scanned data. The spectrum was set as 20 color segments, with the maximum/minimum nominal as ±50 μm and maximum/minimum critical as ±500 μm. The area with the deviations within tolerance limit (max/min nominal: ±50 μm) is represented in green. Regions where the scanned data were larger than the reference data with the magnitude of more than the overtolerance limit (+50 μm) are shown in yellow to red colors, and regions where the scanned data were smaller than the reference model with a magnitude of more than the undertolerance limit (–50 μm) are shown in blue to dark blue. Additionally, to measure the ratio of a deviated region from the reference data, the same software (Geomagic Verify 2015; 3D Systems) was used with a tolerance level of ±50 μm for larger regions (overtolerance) and smaller regions (undertolerance).

### Statistical analysis

IBM SPSS Statistics 21 (IBM SPSS Inc., Armonk, NY) was used for the statistical analysis. The Shapiro‐Wilk test was used for normality test of data, and Levene's test was used for homogeneity of the variance test. Since all data used in this study satisfied these two tests, one‐way ANOVA with Tukey's post hoc test was performed to evaluate the statistical significance of the differences between the groups (significance level: 0.05).

## Results

Concerning trueness (Table 1), there were statistically significant differences among the three groups (*p < *0.05); however, there was no statistically significant difference between stone and SLA models (*p > *0.05). In the comparison of precision among three groups, there was a statistically significant difference among the three groups (*p < *0.05), and there were statistically significant differences between each group (*p < *0.05). The normality and homogeneity of the variance of all used data were confirmed by the Shapiro‐Wilk test and Levene's test (*p > *0.05).

**Table 1:** Trueness and precision of three dental models for complete‐arch model

	Stone (*n = *10)	SLA (*n = *10)	PolyJet (*n = *10)	*p*‐Value
Trueness, RMS (μm)	111.2 ± 3.1^a^	114.3 ± 1.8^a^	124.0 ± 3.7^b^	<0.05*
Precision, RMS (μm)	65.9 ± 8.2^c^	59.6 ± 8.2^b^	41.0 ± 5.8^a^	<0.05*

^*^ *p*‐Value of one‐way ANOVA among three groups is less than 0.05.

^abc^Different superscript letters indicate a significant difference among the means in each row, *p < *0.05 by post hoc Tukey's test.

**Figure 2:**
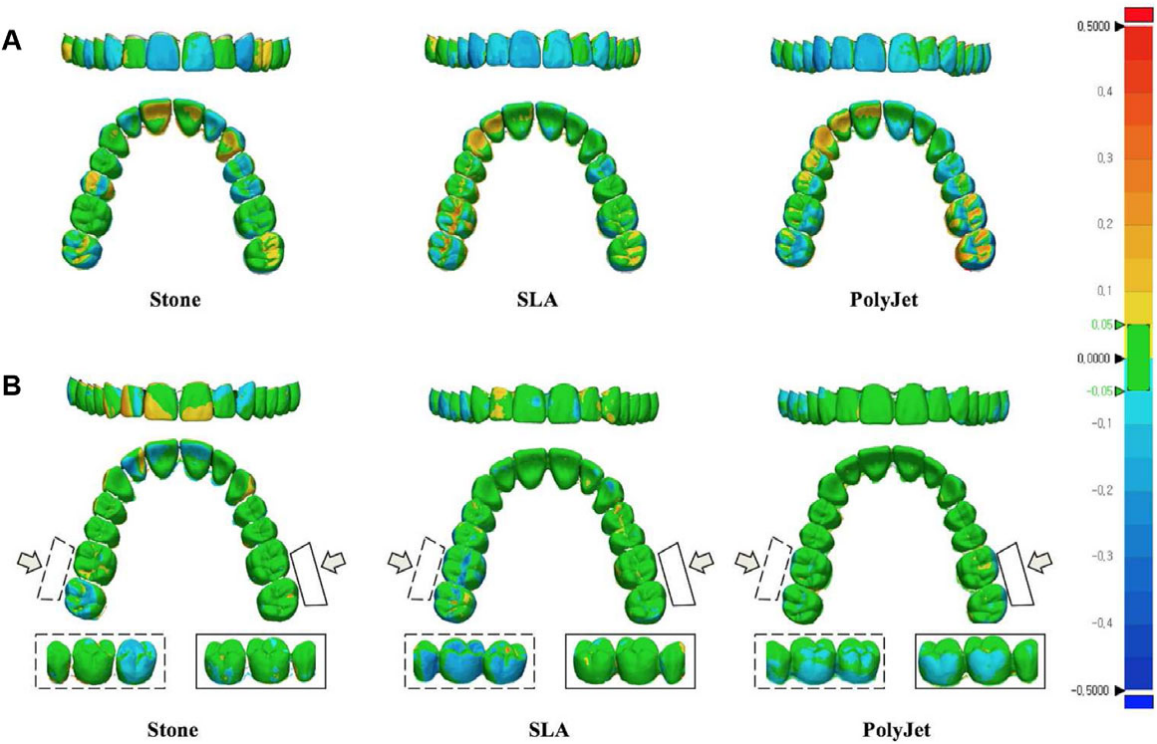
Three‐dimensional deviations between scanned and reference data (A: trueness) and within each tested group (B: precision).

Because all of the deviation images for each model had similar deviation patterns, a representative image for each model was chosen as Figure 2. Figure 2A (trueness) shows that all three groups have blue areas on maxillary anterior regions. AM models have more blue areas than the stone model and show uneven deviations on the occlusal surface of the posterior region. In Figure 2B (precision), all three groups show deviation on the back side of the posterior region (inset images at the bottom of Fig 2), and, especially, AM models show more blue areas on buccal posterior regions.

As for the tolerances (Table 2) measured as the area ratios of the region that are over the upper limit (overtolerance ratio) and are below the lower limit (undertolerance ration), there was a statistically significant difference among the three groups (*p < *0.05); however, no statistically significant difference was observed between the stone and the SLA models in both over‐ and undertolerance ratios (*p > *0.05). Additionally, all three groups presented higher undertolerance than overtolerance ratios. The normality and homogeneity of the variance of all used data were confirmed by the Shapiro‐Wilk test and Levene's test (*p > *0.05).

**Table 2:** Over‐ and undertolerance measures of the three dental models for complete‐arch model

	Stone (*n = *1 0)	SLA (*n = *10)	PolyJet (*n = *1 0)	*p*‐Value
Overtolerance (%)	16.64 ± 2.38^a^	16.85 ± 3.22^a^	23.48 ± 1.65^b^	<0.05*
Undertolerance (%)	16.97 ± 3.08^a^	19.05 ± 3.20^a^	24.36 ± 1.08^b^	<0.05*

^*^ *p*‐Value of one‐way ANOVA among three groups is less than 0.05.

^ab^ Different superscript letters indicate a significant difference among the means in each row, *p < *0.05 by post‐hoc Tukey's test.

## Discussion

Results of this study on the comparison of the trueness and precision between different dental replica models, AM models, and stone models may facilitate the clinical application of AM models using photopolymer materials. From the results, the null hypothesis on trueness that the SLA model had no significant differences compared with the stone model was accepted, but the null hypothesis on precision was rejected.

Linear measurements are frequently used to measure the accuracy of dental replica models, but have limited number of measuring points.^7^, ^11^ Furthermore, since the positions of measuring points on the surface affect the accuracy,^6^, ^12^, ^13^, ^14^ the rough surface of AM models hinders the identification of reference markers on it.^14^ In this study, 3D analysis software was used to reduce this type of error by using multiple overlapping points and to increase the accuracy of measurement. By doing this, the error introduced by the operators could also be minimized.^15^ Furthermore, using 3D deviation image analysis, the deviation ratio was measured, and the regional difference was identified.

In the comparison of trueness, there was no significant difference between the stone and the SLA models, as already shown in various studies.^16^, ^17^, ^18^ In addition, there was no significant difference in the tolerance analysis between these two models (Fig 2A). According to these results, the SLA model appeared to have comparable trueness to the stone model. The comparison between the stone and the PolyJet models showed, however, a significant difference in trueness, which can be explained by the high accuracy material used for the stone models.^19^ However, since previous studies on the trueness of digital models recommended that measurement errors of <200 μm be permitted in a clinical setting, considering that the average trueness of the PolyJet models was 124.0 μm (Table 1), they could potentially be used in a clinical situation.^20^ The SLA model appeared to have better trueness than the PolyJet model, which is possibly explained by the fact that while the SLA model had less polymerization shrinkage from faster multiple polymer photocuring,^21^ the liquid photopolymers used in the PolyJet model suffered more evaporation and more shrinkage than the SLA model.^22^, ^23^

AM models showed an obvious shrinkage pattern in anterior regions (Fig 2A). Since the maxillary anterior region is a morphologically smooth region, the polymers might have contracted more evenly; however, the occlusal surface of the posterior region showed more uneven shrinkage patterns, probably explained by the groove regions.^24^ Also this shrinkage pattern might be affected by the scan path of the intraoral scanner, for which we followed the manufacturer's recommendation.

In terms of precision (Table 1), even though there were significant differences among the three groups, the two AM models showed better precision than the stone model. This might be due to the minimized operator errors made by the AM process.^25^ Although it might be influenced by the impression material and stone preparation settings, we used impression and stone materials of already proven accuracy.^25^, ^26^, ^27^ Between the two AM models, the PolyJet model had the best precision, which can be explained by the fact that the PolyJet process uses photopolymer from a new cartridge every time,^1^ whereas the SLA process reuses nonpolymerized resins, which might introduce errors during the phase change of the liquid resin.^3^ Furthermore, in general (Fig 2B), AM models had more green areas than the stone model, but they had more blue areas in the occlusal surface of the posterior region (i.e., more deviation than the stone model). This result can be explained by a higher density of photopolymers in the posterior region than in the anterior region: more polymer chains in the AM model process might introduce more deviation.^28^ The stone model in Figure 2B seemed to have localized deviation patterns, which are due to random errors in the stone model manufacturing process.^25^

In Table 2, all three groups had higher undertolerance rates than overtolerance rates. The shrinkage of the stone model had been studied previously.^29^, ^30^ Although the ADA specified shrinkage of type IV stone is 0.00% to 0.01%, related studies have reported a wide range of the deviation, from 3^31^ to 35 μm.^32^ As for AM models, biased rates toward undertolerance can be due to the polymerization process using photopolymers.^28^

Another factor influencing accuracy measurements on 3D dental models is the accuracy of the scanner for digital impression.^12^, ^29^ The scanners used in this study appeared to have enough accuracy (the extraoral scanner presented an accuracy of 10 μm, and the intraoral scanner presented a trueness of 45.2 μm, according to manufacturer's documents), and might have had no major effect on the results.^25^, ^27^ Also applying an imaging powder on AM models before the scanning might affect the accuracy of the AM models.

Since this was an in vitro study, its results can be a guide for future in vivo research with more clinical settings. Moreover, research on validation of AM models in patients’ oral cavities will be useful.

## Conclusions

Given the limitations of this study, the following conclusions can be drawn:

1. Although the AM models using photopolymer materials did not show better trueness than the conventional stone models, there was no significant difference between the SLA and the stone models.
2. Concerning precision, the AM models using photopolymer materials presented better results than the conventional stone models.
3. In sum, the AM models using photopolymer materials showed sufficient accuracy for clinical use.
